# Nanopore sequencing provides snapshots of the genetic variation within salmonid alphavirus-3 (SAV3) during an ongoing infection in Atlantic salmon (*Salmo salar*) and brown trout (*Salmo trutta*)

**DOI:** 10.1186/s13567-024-01349-z

**Published:** 2024-09-03

**Authors:** HyeongJin Roh, Kai Ove Skaftnesmo, Dhamotharan Kannimuthu, Abdullah Madhun, Sonal Patel, Bjørn Olav Kvamme, H. Craig Morton, Søren Grove

**Affiliations:** 1 Institute of Marine Research, Nordnes, PO Box 1870, 5817 Bergen, Norway; 2 Norwegian Veterinary Institute, Bergen, Norway

**Keywords:** Salmonid alphavirus (SAV), nanopore sequencing, viral mutation, E2 gene, spike protein, viral heterogeneity

## Abstract

Frequent RNA virus mutations raise concerns about evolving virulent variants. The purpose of this study was to investigate genetic variation in salmonid alphavirus-3 (SAV3) over the course of an experimental infection in Atlantic salmon and brown trout. Atlantic salmon and brown trout parr were infected using a cohabitation challenge, and heart samples were collected for analysis of the SAV3 genome at 2-, 4- and 8-weeks post-challenge. PCR was used to amplify eight overlapping amplicons covering 98.8% of the SAV3 genome. The amplicons were subsequently sequenced using the Nanopore platform. Nanopore sequencing identified a multitude of single nucleotide variants (SNVs) and deletions. The variation was widespread across the SAV3 genome in samples from both species. Mostly, specific SNVs were observed in single fish at some sampling time points, but two relatively frequent (i.e., major) SNVs were observed in two out of four fish within the same experimental group. Two other, less frequent (i.e., minor) SNVs only showed an increase in frequency in brown trout. Nanopore reads were de novo clustered using a 99% sequence identity threshold. For each amplicon, a number of variant clusters were observed that were defined by relatively large deletions. Nonmetric multidimensional scaling analysis integrating the cluster data for eight amplicons indicated that late in infection, SAV3 genomes isolated from brown trout had greater variation than those from Atlantic salmon. The sequencing methods and bioinformatics pipeline presented in this study provide an approach to investigate the composition of genetic diversity during viral infections.

**Supplementary Information:**

The online version contains supplementary material available at 10.1186/s13567-024-01349-z.

## Introduction

The emergence of new viral strains with increased virulence is of great concern to the aquaculture sector. Salmonid alphavirus (SAV) is the causative agent of pancreas disease (PD) in Atlantic salmon (*Salmo salar*) and of sleeping disease (SD) in rainbow trout (*Oncorhynchus mykiss*). SAV is an enveloped, spherical, single-stranded positive-sense RNA virus with a diameter of ~70 nm belonging to the *Togaviridae* family. The SAV genome is approximately 12 kb long and comprises two open reading frames (ORF1 and ORF2) that both encode polyproteins [1]. ORF1 encodes four nonstructural proteins (nsP1, nsP2, nsP3, and nsP4) that are required for RNA synthesis [2]. Like for other alphaviruses, SAV ORF2 likely encodes six structural proteins, i.e., C, E2, E3, 6 k, E1 and TF, where C is the capsid protein and E1, E2 and E3 are constituents of the heterotrimeric spike proteins in the envelope [3, 4]. 6 k is an ion channel protein [5], whereas the TransFrame (TF) protein, known from several alphaviruses, is produced by a ribosomal –1 frameshift in 6 k. The TF protein has the same N-terminus as 6 k but a unique C-terminus, which may be relevant to virion stability, antigenicity, fusion, and tropism [4, 6].

Since SAV was first identified in 1995, at least six subtypes have been described based on nucleotide sequence analysis of nsP3 and E2 [7, 8]. More recently, the existence of a seventh genotype has been proposed based on an SAV isolate from Ballan wrasse (*Labrus bergylta*) [3]. The SAV subtypes show differences in geographical distribution, host range, and clinical manifestations [1, 9, 10]. SAV1 (salmon pancreas disease virus; SPDV) and SAV2 (sleeping disease virus; SDV) were characterized as two separate subtypes from approximately 1999–2000 [11, 12]. The SAV3 subtype (Norwegian salmonid alphavirus; NSAV) was first characterized by Hodneland et al. [13]. Over the whole genome, the subtypes have been shown to share ~86–96% genetic identity [3, 13].

Gallagher et al. [8] reported SAV sequencing data suggesting that individual farmed fish may become coinfected with different SAV subtypes. Infection of a host with two or more viral subtypes may be a basis for viral genetic changes via recombination. Similarly, a single SAV subtype transmitted from one host species or region to another may undergo genetic changes during adaptation [8, 14, 15]. RNA viruses generally have high mutation rates of between ~10^–6^ and 10^–4^ substitutions per nucleotide site per cell infection. A previous study estimated the SAV substitution rate to be approximately 1.70 (± 1.03) × 10^–4^ nt substitution/site/year [16]. A more recent study of the genome-wide substitution rate for SAV3 estimated 7.351 × 10^–5^ substitutions per site per year, with a 95% highest posterior density range of 5.33 × 10^–5^–9.994 × 10^–5^ [17]. In addition, there is evidence that SAV can frequently undergo mutations and deletions even within a single host [8, 18]. Petterson et al. [18] reported that many genome deletions are generated during natural SAV infection, and subsequent verification of frequent deletion mutations was achieved using nanopore sequencing methods [17]. The low fidelity of the RNA-dependent RNA polymerase (RdRp) and the high incidence of recombination via template switching during replication both contribute to this high mutation rate [19–21]. The copy choice model is a widely accepted mechanistic model for viral recombination and is particularly relevant for single-stranded positive-sense RNA viruses such as SAV [22, 23]. In an infected cell, erroneous replication may produce considerable variation in the virus genome sequence and thus in the expressed viral proteins. In addition to this type of variation, selective pressure may also lead to “intracellular adaptations” that improve viral fitness in a particular host cell environment, including adaptations to codon and codon pair usage, improved suppression of the IFNα/β response and more [24]. Viral particles exiting infected cells may differ in the amino acid (aa) sequence of their capsid and spike proteins, leading to possible changes in their receptor binding affinities and specificities and hence potentially to changes in cell, tissue and host tropism. Virus particles with altered protein sequences may also be less prone to recognition by specific antibodies. With such variation and the inferred potential differences in viral function, fitness and adaptability, the viral consensus sequence may be insufficient to characterize a virus. Instead, the variation can be better understood as a mutant spectrum or quasispecies, which may provide a better definition of wild-type virus [25].

Long-read deep sequencing technologies, such as single-molecule real-time sequencing by Pacific Biosciences and Oxford Nanopore, have significantly contributed to the understanding and profiling of genetic variations in pathogens [26–29]. In particular, Oxford Nanopore long-read sequencing technology has proven useful for identifying new SAV genotypes and for profiling SAV mutation sites [3, 8, 30]. Until recently, a prevailing issue with long-read sequencing platforms has been the inherent low base-calling accuracy [31], which may lead to the misidentification of mutations in individual nanopore reads. Several methods have been proposed to complement and overcome this limitation. Gallagher et al. [32] demonstrated that sequencing errors generated from the Oxford nanopore platform can be minimized by achieving a sufficient sequencing depth. They found that a sequencing depth of more than 50 × was sufficient to accurately sequence the SAV genome. Aligning long reads to a consensus sequence is a standard pipeline for identifying single nucleotide polymorphisms (SNPs) and structural variants. However, the relatively high error rate in individual reads can pose a challenge in distinguishing rare minor variants from within the cloud of nonvariant reads. As an alternative, unique molecular identifiers (UMIs) have been utilized to address sequencing errors, but other technical challenges, such as accurate titration of input templates and sequencing depth, remain a challenge [33, 34]. In the most recent advancements, due to improvements in the chemistry of sequencing library preparation kits, the structural and functional properties of nanopores, and recent changes in base-calling algorithms, the accuracy of each raw read can now be over 99.9% (> Q30) with the duplex basecalling algorithm [35]. By excluding reads found in low numbers, likely representing random sequencing errors, the sequencing fidelity of reads included in the analysis can be increased.

With such high accuracy of single reads, sequence diversity can be profiled by de novo clustering using high thresholds of sequence identity, a technique that is widely applied in microbiome studies from PCR amplicons. In such studies, sequence reads from PCR amplicons (e.g., from the 16S or 18S rRNA gene) can be clustered and classified as operational taxonomic units (OTUs) based on sequence identity [36, 37]. Alongside the advantage of amplicon clustering, the high accuracy of single long reads enables the relatively precise profiling of minor variants within a sample. In other words, it allows for both the identification of genetic variation within a sample and de novo assembly of multiple complete genomes for viral variants, strains, and/or quasispecies within a sample. In this study, nanopore sequence reads were clustered based on sharing at least 99% sequence identity. The cluster containing the largest number of reads was designated the “major cluster”, while clusters with fewer sequence reads were defined as “minor clusters”. The consensus that can be generated from each cluster may provide an overview of the most frequent variants present in the analysed samples. In this study, we aimed to 1) develop an SAV3 variant identification method within a sample using high-accuracy nanopore reads; 2) identify major and minor SAV3 variants that arise during an active infection; and 3) explore potential genetic variations that occur when SAV3 infects either Atlantic salmon or brown trout.

## Materials and methods

### Fish and viral challenge

Atlantic salmon and brown trout were reared at the Institute of Marine Research (IMR), Research Station in Matre (Masfjorden, Norway). Prior to viral challenge, the fish were transported to IMRs fish disease laboratories in Bergen (Norway). The salmon and trout were acclimated in 400 L tanks supplied with freshwater at a flow rate of approximately 400 L h^−1^. Commercial feed was provided twice daily, and the water temperature was maintained at 10–12 °C. The photoperiod was maintained at 12 h light and 12 h dark during both the acclimation and experiment. Viral challenge was performed as a cohabitation challenge. In brief, naïve salmon shedder fish were injected intramuscularly with a 2 × 50 µL of 1 × 10^4^ TCID_50_ mL^−1^ SAV3 inoculum [38]. The virus was propagated in CHH-1 cells, and passage 3 of the virus was used in this trial. The shedder fish were marked by the adipose fin clipping method for selective sampling of cohabitant fish during the subsequent sampling period. Then, 30 salmon shedders and 70 naïve salmon or trout were transferred to 250 L experimental tanks where they remained for the duration of the cohabitation challenge experiment. At 2, 4, and 8 weeks after cohabitation started, sixteen cohabitation fish of each species were euthanized using an overdose of Benzocaine (160 mg L^−1^; Apotekproduksjon AS, Norway). Sampling was performed at 2-, 4-, and 8-weeks post-challenge (wpc), producing six experimental groups consisting of specific combinations of sampling time points and fish species (2wpc_Salmon, 4wpc_Salmon, 8wpc_Salmon, 2wpc_Trout, 4wpc_Trout, and 8wpc_Trout). Hearts were dissected from all the fish, transferred to RNALater (Ambion, TX, USA) and stored at −80 °C until further analysis. All experiments involving live animals were approved by the Norwegian Food Safety Authority (FOTS approval number 11260).

### RNA extraction and quantitative PCR (qPCR)

Total RNA was extracted from the heart following the standard protocol of the Promega ReliaPrep^™^ simply RNA HT 384 kit (Promega, WI, USA) on a Biomek 4000 Laboratory Automated Workstation (Beckman Coulter, CA, USA). The total RNA concentration was quantified using a NanoDrop^™^1000 spectrophotometer (Thermo Scientific, MA, USA), and the RNA samples were diluted to 100 ng µL^−1^ using a Biomek 4000 Laboratory Automated Workstation (Beckman Coulter, CA, USA). Quantitative RT-PCR was conducted using the AgPath-ID One Step RT-PCR kit (ThermoFisher, MA, USA) according to the manufacturer’s instructions with primers targeting the SAV3 nsP1 gene (F: 5′-CCGGCCCTGAACCAGTT-3′; R: 5′-GTAGCCAAGTGGGAGAAAGCT-3′ and probe: 6FAM-TCGAAGTGGTGGCCAG-MGBNFQ)[39]. Briefly, 200 ng of total RNA was added to a reaction mixture containing 400 nM forward and reverse primers and 160 nM probe in a total volume of 10 µL on a 384-well plate [39]. The qPCR protocol included reverse transcription (1 cycle: 45 °C/10 min), predenaturation (1 cycle: 95 °C/10 min), 40 cycles of amplification (95 °C/15 s and 60 °C/45 s) and fluorescence detection using a QuantStudio 5 real-time PCR system (Applied Biosystems, MA, USA).

### Nanopore sequencing library preparation

**Figure 1:**
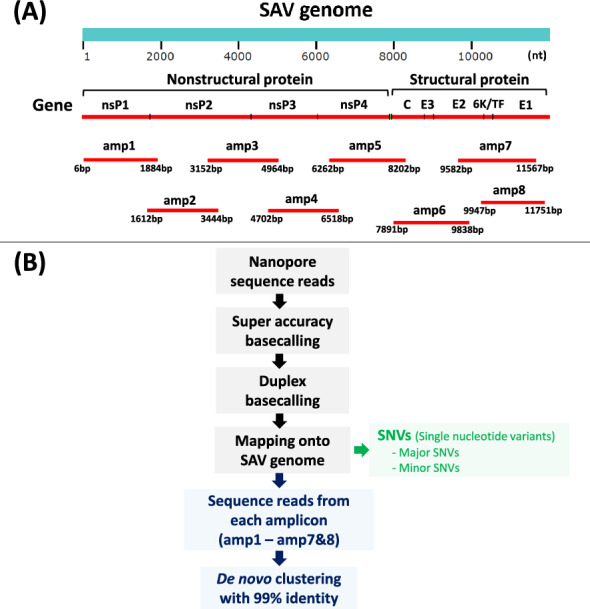
**The SAV3 genome, amplicon details and the bioinformatic protocol applied in the study. A** The ~12 kb SAV3 genome encodes four nonstructural proteins (nsP1-4) and five structural proteins (C-E1), and the eight overlapping amplicons (amp1-8) cover ~98.8% of its length. **B** Schematic diagram of the bioinformatic approaches used in the study. Gray boxes: from nanopore sequencing of amplicons to mapped SAV3 reads; Green box: identification of single nucleotide variants (SNVs); Blue boxes: workflow to identify consensus clusters inferred from SAV3 reads sharing at least 99% sequence identity.

Only heart samples with Ct values below 35 were included for analysis via nanopore sequencing. A total of 22 heart samples from salmon and trout at 2, 4, and 8 wpc were included in this experiment. Each experimental group (i.e., fish species at a specific sampling time point) included 3–4 samples, given the maximum of 24 barcodes available in the nanopore sequencing library used in this study (Additional file 1). From each sample, 1 µg of total RNA was added to a total of 10 µL of cDNA reaction mix containing 10X SuperScript reverse transcriptase, 5X VILO reaction and random hexamers (SuperScript VILO cDNA synthesis kit (Invitrogen, MA, USA)). The cDNA mixture was then sequentially incubated at the following conditions: 25 °C for 10 min, 42 °C for 60 min, 50 °C for 30 min, and 85 °C for 5 min. For each sample, eight sets of PCR primers were used to produce eight amplicons (amplicon1—amplicon8; amp1—amp8) that covered most of the SAV genome (Figure 1A; Additional file 2). Briefly, the PCR mixture was prepared using the following components: 2 µL of 5X Q5 reaction buffer, 0.2 µL of 10 mM dNTPs, 0.1 µL of Q5 hot-start DNA polymerase (20 units mL^−1^), primers (forward and reverse; 5 µM), 1 µL of cDNA (synthesized from 100 ng of total RNA), and DNase-free water up to 10 µL. The PCR conditions were as follows: 1 cycle of denaturation (98 °C for 30 s), 35 cycles of amplification (98 °C for 10 s, 62 °C for 30 s, and 72 °C for 3 min), and 1 cycle of post-extension (72 °C for 8 min). Amplicons were cleaned using AMPure XP beads according to the manufacturer’s guidelines (Beckman Coulter, CA, USA). Blunt end repair and DNA ligation were carried out using the NEBNext End Repair Module and NEBNext Ligation Sequencing Kit (NEBNext, MA, USA). A Native Barcoding Kit 24 (Q20 + and duplex enabled, Oxford Nanopore, UK) was used to obtain a unique barcode for all eight amplicons from each sample. All the barcoded samples were then pooled together and sequenced using a MinION flow cell (R10.4, Oxford Nanopore, UK).

### Bioinformatics

#### Basecalling

Basecalling was performed using the GPU-enabled guppy6.06 basecaller with the super accuracy configuration dna_r10.4_e8.1_sup.cfg. Since the accuracy of the raw reads is important for downstream variant calling analyses, we further implemented the newer duplex basecalling capability introduced by the Oxford Nanopore Company (Oxford Nanopore, UK). Duplex tools were used to identify duplex pairs. The guppy duplex basecalling command was then executed with the super accuracy configuration (dna_r10.4_e8.1_sup.cfg), and the duplex pair information identified in the prior step was used as input. The flags “*–barcode_kits “SQK-NBD112-24”–trim_barcodes –trim_adapters –trim_strategy dna –require_barcodes_both_ends*” were included in this command to ensure proper demultiplexing and trimming of adapter sequences.

#### Single nucleotide variant (SNV) identification

**Figure 2:**
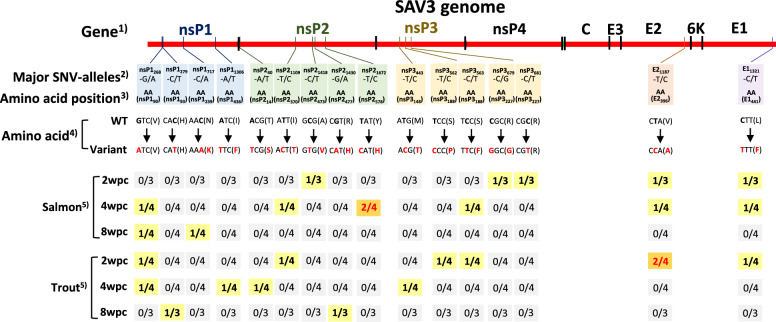
**The incidence of major SNV-alleles in the experimental groups.** The individual locations of each SNV are marked on the SAV3 genome.The ratio of fish with major SNV-alleles in the various experimental groups (2wpc_Salmon, 4wpc_Salmon, 8wpc_Salmon, 2wpc_Trout, 4wpc_Trout, and 8wpc_Trout). **1** The positions of each gene on the SAV3 genome, **2** details of the major SNV-allele, **3** amino acid position numbering for each protein, and **4** resulting changes in amino acids, i.e., from WT (2wpc_Salmon consensus genome) to variant (changes shown in red), **5** experimental groups (i.e., fish species at specific sampling time points). Each experimental group in which one fish was shown to have an SNV is shown in bold black numbers and yellow. Each experimental group, where two fish were shown to have a specific SNV-allele, is shown in red bold numbers and orange.

To identify single nucleotide variants (SNVs) (Table 1) occurring in salmon samples at 4 and 8 wpc and all trout samples, a consensus genome was constructed from the reads from the salmon samples at 2 wpc. Briefly, the sequence reads from the 2wpc_Salmon experimental group were mapped onto the published SAV3 genome (SAV3-2-MR/10 isolate; GenBank accession: KC122926), after which Tablet (ver. 1.21.02.08) [40] was used to generate the “2wpc consensus genome”. All variant analyses were conducted using the 2wpc consensus genome. The FastQ files for each sample, identified by the barcodes, were mapped onto the 2wpc consensus genome using Bowtie2 with the *“very sensitive* option” [41]. The SAM file was converted to a sorted BAM file using samtools, and the variant calling file (vcf) was produced using BCFtools call with the command “-m” or “-mv" [42, 43]. The terminology related to the analysis of SNVs conducted in this study is defined in Table 1. Excluding primer binding site sequences, SNVs were identified using the variant calling command with the “-mv” option. Any of the three possible nucleotides that differed from the nucleotide in the reference genome at a polymorphic site were defined as “SNV alleles” (Table 1). SNV-alleles with an SNV allele frequency ranging from 5–60% were considered minor SNV-alleles while SNV-alleles with an SNV-allele_freq_ above 60% were considered major (Table 1). For each sampling time point and fish species (i.e., experimental group), the number of major SNV-alleles was counted (Figure 2).

**Table 1:** **Definition of terminologies for the analysis of single nucleotide variants (SNVs) and sequence read clustering investigated in this study**

Purpose	Term	Definition
Analysis of the single nucleotide variant (SNV)	Single nucleotide variant (SNV)	At any given polymorphic site, there are three possible alleles that differ from the nucleotide in the reference genome
	SNV frequency (SNV_freq_)	The fraction of total reads with given SNVs (Number of reads with SNVs/total number of reads) × 100
	Major SNV	An SNV with SNV_*freq*_ ≥ 60% in at least one experimental group
	Minor SNV	An SNV with SNV_*freq*_ between 5 - 60% in at least one experimental group
	SNV-allele	Any of the three possible nucleotides at a polymorphic site that differ from the nucleotide in the reference genome
	SNV-allele frequency (SNV-allele_freq_)	The fraction of total reads with given SNV-allele. (Number of reads with a given SNV-allele/total number of reads) x 100
	Major SNV-allele	An SNV allele with SNV-allele_*freq*_ ≥ 60% in at least one experimental group
	Minor SNV-allele	An SNV allele with SNV-allele_*freq*_ between 5 - 60% in at least one experimental group
	SNV-Gene_XYZ_	SNV occuring at site/position XYZ from the starting point of each gene
	SNV-Gene_XYZ_-A2/A1	SNV occurring at site/position XYZ from the starting point of each gene with nucleotide A2 (Allele2; reference allele) and A1 (allele1; variant allele)
Analysis of sequence reads clustering for each amplicon	AmpX-cluster	Cluster of reads produced from clustering analysis of reads mapping to amplicon X (X=1-8)
	Proportion of reads in Xcluster	The read counts assigned to the X cluster were divided into total read counts in the corresponding amplicon from a sample.
	Cluster consensus	The consensus sequence inferred from the reads belonging to a given cluster
	Major cluster	The cluster at each amplicon that has the most sequenced reads > 70%
	Minor cluster	All the clusters for each amplicon except for the major cluster

#### Identification *of major* and minor SAV3 cluster(s) in each amplicon

For each sample, all the sequence reads in the FastQ files were mapped onto each of the eight individual amplicons using Bowtie2 with the same options as described in the subsection “Single nucleotide variant (SNV) identification”. The reads from amplicon (amp) 7 and amp8 were pooled together for clustering because the amplicons overlapped somewhat (Figure 1). Antisense reads in the sets were transformed to complementary sense reads using FASTX-Toolkit [44, 45]. The reads from each amplicon were de novo clustered (i.e., amp1-cluster to amp8-cluster) using qiime2 and a 99% sequence identity threshold [46]. In detail, the sample information and FastQ files were processed (“tools” option with the flags “– type SampleData[SequencesWithQuality]” and “–input-format SingleEndFastqManifestPhred33V2”) to.qza file using qiime2. Then, the individual sequences and table files were extracted with the flag “vsearch dereplicate-sequences”, and finally, de novo clustering was carried out through “vsearch cluster-features-de-novo”, with the flag “–p-perc-identity 0.99″. Only reads not shorter than 90% of the amplicon length were included in the clustering, and only clusters that contained at least 0.5% of all reads for the given amplicon were used for further analysis. For each amplicon, the clusters passing the above criteria were then aligned, and phylogenetic trees were produced using the maximum likelihood phylogenetic method with 1000 bootstrap replicates in MEGA11 [47, 48].

### Visualization of the location of selected deletions and SNVs in the SAV3 spike protein

The amino acid sequences for E1, E2, and E3 from the 2wpc_consensus genome were used. The SAV3 spike protein structure was modelled using homology modelling in SWISS-MODEL in automated mode [49]. The 3D structure of the SAV3 spike protein model was visualized using PyMOL software [50, 51]. The predicted 3D structure was used to visualize the location of the deletions observed in those of the minor clusters that contained at least 10% of the reads (i.e., a proportion > 10%). Additionally, the sites with nonsynonymous minor or major SNVs are also shown in the 3D structure.

### Statistical analysis

Duncan’s HSD one-way ANOVA was used for the statistical analysis of Ct values and relative cluster size data. Welch’s two-sample t test was used for the SNV_freq_ and SNV-allele_freq_ analyses. The threshold of the *p* value was set to less than 0.05. All the statistical analyses were carried out using the “haven” library in R [52]. The statistical significance of the frequency of major SNV-alleles compared to the amino acid composition of the SAV3 2wpc_Salmon consensus genome was confirmed using chi-square testing in R.

## Results

### Viral load

The viral load in the samples included in the sequencing was assessed using qPCR. For Atlantic salmon, the mean Ct values were 28.9 ± 6.3, 22.6 ± 3.9, and 26.8 ± 0.4 at 2, 4, and 8 wpc, respectively. For trout, the parallel Ct values were 25.9 ± 4.0, 21.9 ± 0.8, and 33.4 ± 1.0, respectively. Significant differences in viral load measured by the Ct values between species were observed at 8 wpc (Additional file 3).

### Nanopore sequencing

More than five million raw nanopore reads were contained in the Fast5 file obtained from the sequencing experiment using a single R10.4 nanopore flow cell. The Fast5 file was converted to nucleotide sequences using guppy 6.06 with the super accuracy base-calling algorithm, resulting in 5,278,494 reads with a median Phred quality score of 16.412 (equivalent to ~97.72% estimated accuracy). Using the duplex basecalling algorithm, we obtained 166740 reads that passed the more rigorous filtering implemented in this method, corresponding to less than 3.2% of the total reads. However, the median Phred quality score was much greater at 24.109, equivalent to ~99.61% estimated accuracy (mean Phred quality score ± standard deviation = 25.116 ± 7.392). Among them, 97,761 reads could be properly identified by the barcode. This study exclusively employed high-quality sequence reads that were accurately identified by barcodes after duplex basecalling. On average, ~50% of the high-quality sequence reads (45,318 out of 97,791 reads) were successfully mapped onto the reference genome (Additional file 1). Upon examination of unmapped sequences, sequences harboring high similarity to SAV were identified but were characterized by the presence of sequence transpositions, inversions, large insertions, or deletions. Whether these unmapped sequences were PCR artefacts or originated from viral variation was not examined in this study.

### Major and minor mutation changes in SAV

**Figure 3:**
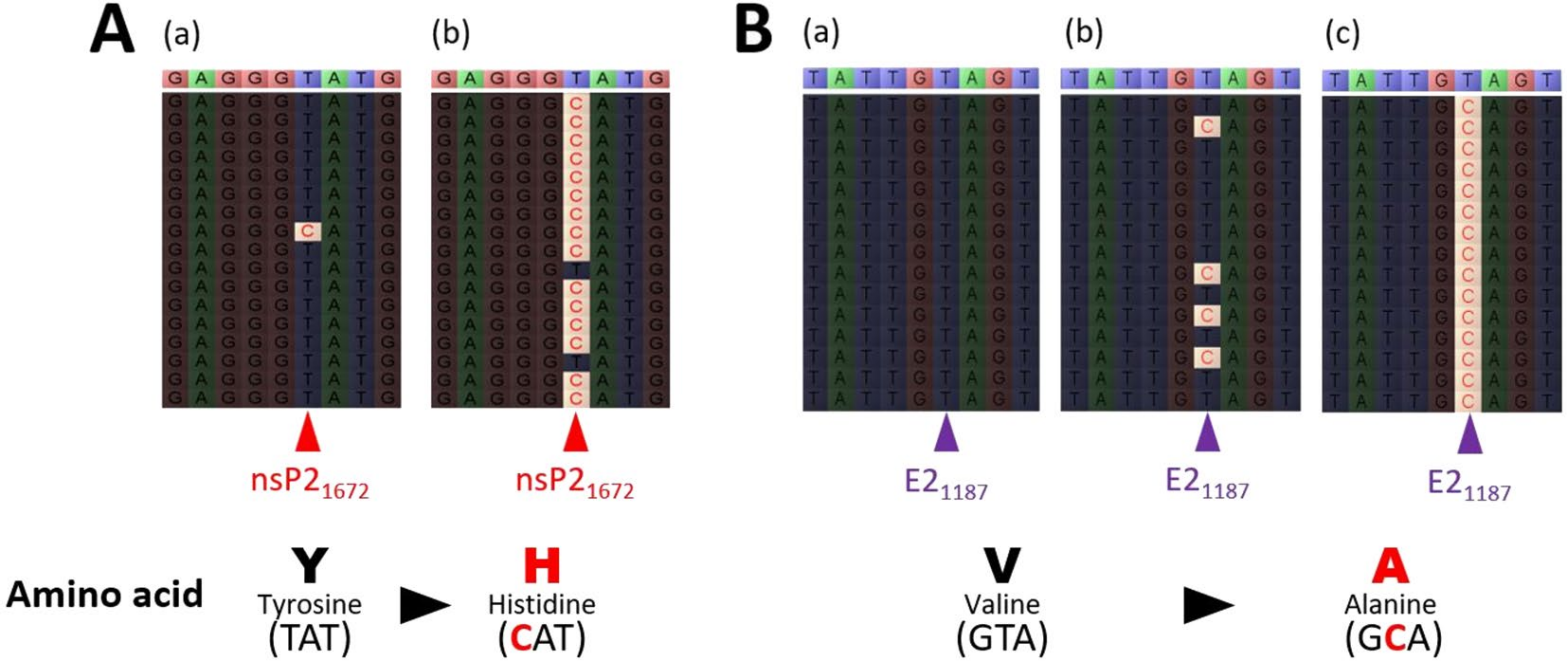
**Examples illustrating the difference in the frequency of selected SNV-alleles in individual fish/samples.** For five fish (**A**-a to **B**-c), sequence reads were aligned against the 2wpc_Salmon consensus genome sequence (upper, coloured sequence). The nucleotides in the reads that differed from the corresponding consensus nucleotides are shown in red. A) Comparison of reads from two salmon samples at 2 wpc centred around the major SNV-allele nsP2_1672_-T/C. There is a distinct difference in the frequency of C in the nucleotide site nsP2_1672_ between (fish) **A**-a and (fish) **A**-b. **B**) Comparison of reads from two trout (**B**-a and **B**-c) and one salmon (**B**-b) sampled at 2 wpc, centred around the major SNV-allele, E2_1187_-T/C. There is a distinct difference in the frequency of C in the nucleotide site E2_1187_. Both major SNV-alleles lead to nonsynonymous changes in codons.

**Figure 4:**
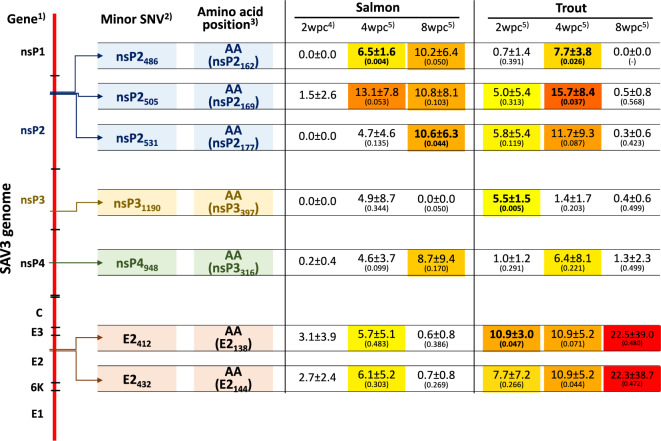
** Ocurrence of minor SNVs in the experimental groups.** A total of 7 SNVs were identified as minor, as they had an SNV_freq_ between 5 and 60% in at least one experimental group. The locations of minor SNVs within the SAV3 genome are shown here. For each minor SNV, a Welch's t test was used to compare the frequencies between the experimental groups and the 2wpc_Salmon consensus genome. **1** The positions of each gene in the SAV3 genome, **2** details of the minor SNVs, **3** amino acid position numbering for each protein, **4** SNV_freq_  of the minor SNVs in the 2wpc_Salmon consensus genome, and **5** SNV_freq_ of the minor SNVs in the experimental groups. The numbers inside brackets show p values from Welch’s t test comparing the SNV frequency in the experimental group with that of the 2wpc_Salmon consensus genome (bold letters indicate P values less than 0.05). The SNVs highlighted with a background color range from yellow to red represent SNV_freq_ values ranging from 5% (yellow) to the highest value (red), with the color intensifying progressively as the values increase. Detailed information on the minor SNVs in the experimental groups is provided in Additional file 4.

Among the 22 samples, a total of 16 major SNV-alleles were identified in this study, and some of the major SNV-alleles were present in multiple samples (Figures 2, 3). Most of these major SNV-alleles appeared to be randomly distributed across the sampling time points and between fish species. However, two major, nonsynonymous SNV-alleles were identified in two out of four fish (50%) in the same experimental group. These mutations, which are located in nsP2 (SNV-nsP2_3414_-T/C) and E2 (SNV-E2_1187_-T/C), resulted in changes from tyrosine to histidine and valine to alanine, respectively (Figure 3). We also noted that while arginine constituted only 6.3% (248/3906) of the amino acids in the 2wpc_Salmon consensus genome, 18.8% (3/16) of the major SNVs occurred in codons for arginine (Table 2). Arginine codons, therefore, were the site of major SNVs three times more frequently than would be expected based on their relative frequency in the genome (*P* = 0.0431). The remaining 19 amino acids did not harbor major SNVs at a frequency that was significantly higher or lower than their frequency within the 2wpc_Salmon consensus genome (Table 2). We also identified 7 minor SNV-alleles distributed in both nonstructural and structural genes (Figure 4, Additional file 4). Most of the minor SNV-alleles resulted in nonsynonymous mutations. The trout group tended to show more frequent changes than did the salmon group, especially in the E2 gene. In the trout experimental groups, the two minor SNV-alleles, SNV-E2_412_ and SNV-E2_432_, increased in SNV_freq_ during the experiment. There was a distinctly greater proportion of SNV-E2_412_-T/C. For SNV-E2_432_, two specific variants, both of which produce a glutamic acid (E) to aspartic acid (D) change (SNV-E2_432_-G/T and SNV-E2_432_-G/C), had a distinct, though not significant, increase in proportion (Additional file 4).

**Table 2:** **The occurrence of major SNV-alleles in codons for amino acids**

Amino acid (aa)	Overall frequency of aa in SAV3 genome	Frequency by which major SNV-alleles occur in the aa^1^	Relative frequency^2^	*P* value^3^
Arginine (R)	6.3% (248/3906)	18.8% (3/16)	3.0	**0.0431**
Isoleucine (I)	4.1% (159/3906)	12.5% (2/16)	3.1	0.0899
Methionine (M)	2.0% (77/3906)	6.3% (1/16)	3.2	0.2212
Histidine (H)	2.6% (103/3906)	6.3% (1/16)	2.4	0.3694
Serine (S)	6.9% (270/3906)	12.5% (2/16)	1.8	0.3800
Tyrosine (Y)	3.3% (130/3906)	6.3% (1/16)	1.9	0.5163
Asparagine (N)	3.5% (137/3906)	6.3% (1/16)	1.8	0.5524
Alanine (A)	10.1% (395/3906)	6.3% (1/16)	0.6	0.6068
Valine (V)	9.3% (363/3906)	12.5% (2/16)	1.3	0.6595
Leucine (L)	7.6% (295/3906)	6.3% (1/16)	0.8	0.8440
Threonine (T)	7.3% (284/3906)	6.3% (1/16)	0.9	0.8753
Aspartic acid (D)	5.6% (218/3906)	0% (0/16)	–	
Cysteine (C)	2.7% (104/3906)	0% (0/16)	–	
Glutamine (Q)	2.9% (115/3906)	0% (0/16)	–	
Glutamic acid (E)	5.0% (196/3906)	0% (0/16)	–	
Glycine (G)	6.5% (254/3906)	0% (0/16)	–	
Lysine (K)	5.1% (199/3906)	0% (0/16)	–	
Phenylalanine (F)	2.7% (106/3906)	0% (0/16)	–	
Proline (P)	5.5% (216/3906)	0% (0/16)	–	
Tryptophan (W)	0.9% (37/3906)	0% (0/16)	–	

A comparison of the frequency of the 20 amino acid codons in the SAV3 2wpc_Salmon consensus genome with the frequency of major SNV-alleles occurring in a codon suggested that the arginine codon harbors a major SNV-allele with a threefold greater relative frequency.

^1^Calculated as the number of times a major SNV-allele is harbored in a given specific aa codon divided by the total number of major SNV-alleles.

^2^The relative mutation frequency is calculated by dividing the frequency by which major SNV-alleles occur in a given amino acid (column 3) by the overall frequency of the amino acid in the SAV3 genome (column 2).

^3^Results from a chi-square test of the ratios in column 2 and column 3. The numbers in bold indicate *p* values lower than 0.05.

### Amplicon clusters and phylogenetic analysis

**Figure 5:**
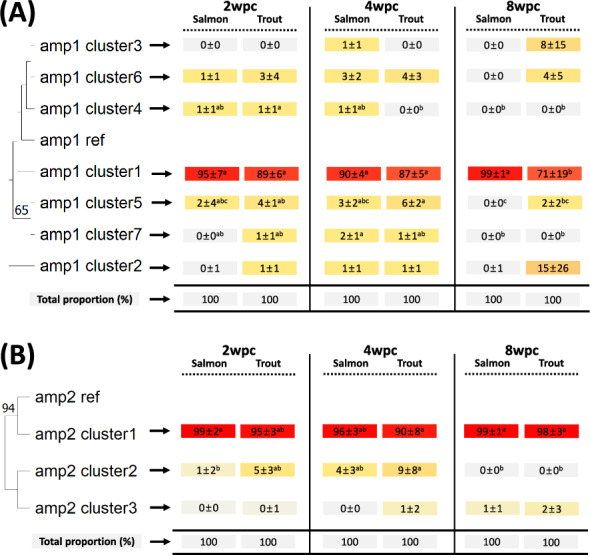
**Phylogenetic tree of the amp1 and amp2 clusters. **The maximum likelihood algorithm was used to construct a phylogenetic tree of the identified clusters from the amplicons amp1 (**A**) and amp2 (**B**) (left side). The numbers (above 50%) near each branch indicate bootstrap values out of 1000 replications. The table on the right side shows the proportion of reads in each identified cluster (proportion mean ± standard deviation (SD)) for each experimental group (i.e., fish species at a specific sampling time point). The color gradient from gray to red indicates the proportion of reads in each cluster. For each cluster, the proportion of reads was compared between experimental groups using Duncan’s HSD one-way ANOVA. Different superscripted letters indicate statistically significant differences (*P* value < 0.05).

**Figure 6:**
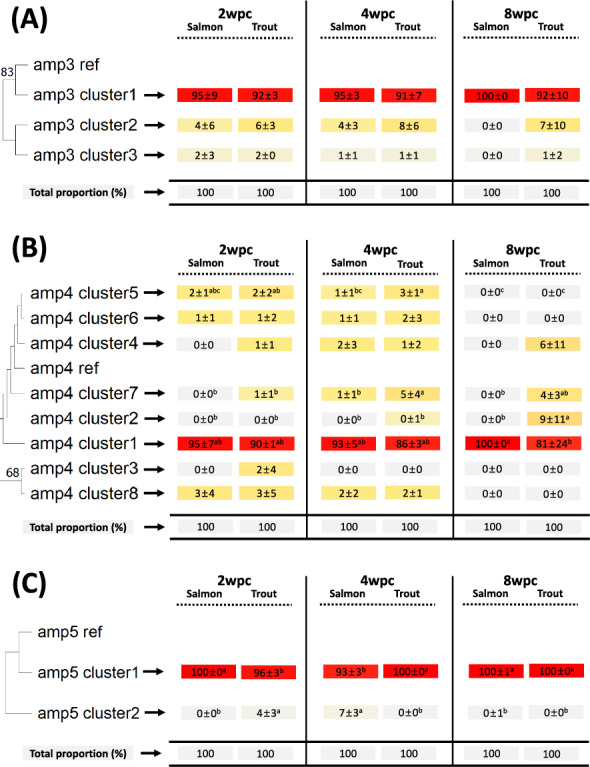
**Phylogenetic tree of the amp3, amp4, and amp5 clusters**. The maximum likelihood algorithm was used to construct a phylogenetic tree of the identified clusters from the amplicons amp3 (**A**), amp4 (**B**), and amp5 (**C**) (left side). The numbers (above 50%) near each branch indicate bootstrap values out of 1000 replications. The table on the right side shows the proportion of reads in each identified cluster (proportion mean ± standard deviation (SD)) for each experimental group (i.e., fish species at a specific sampling time point). The color gradient from gray to red indicates the proportion of reads in each cluster. For each cluster, the proportion of reads was compared between experimental groups using Duncan’s HSD one-way ANOVA. Different superscripted letters indicate statistically significant differences (P value < 0.05).

**Figure 7:**
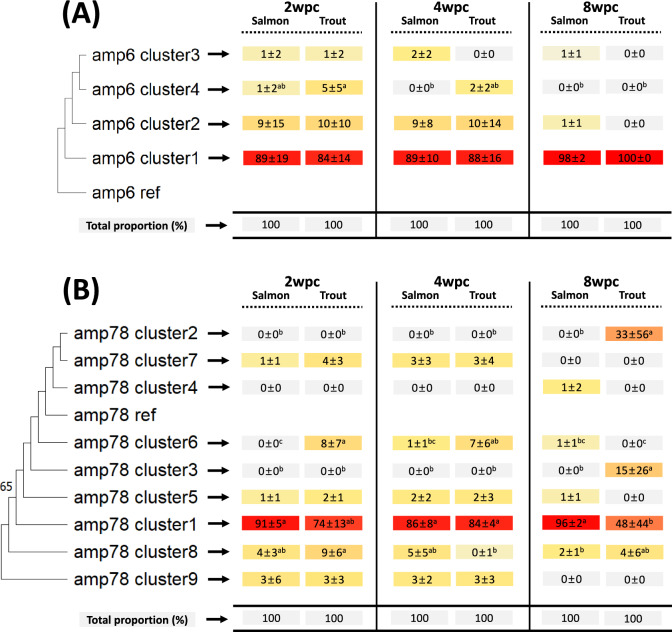
**Phylogenetic tree of the amp6 and amp78 clusters.** The maximum likelihood algorithm was used to construct a phylogenetic tree of the identified clusters from the amplicons amp6 (**A**) and amp78 (**B**) (left side). The numbers (above 50%) near each branch indicate bootstrap values out of 1000 replications. The table on the right side shows the proportion of reads in each identified cluster (proportion mean ± standard deviation (SD)) for each experimental group (i.e., fish species at a specific sampling time point). The color gradient from gray to red indicates the proportion of reads in each cluster. For each cluster, the proportion of reads was compared between experimental groups using Duncan’s HSD one-way ANOVA. Different superscripted letters indicate statistically significant differences (*P* value < 0.05).

Through de novo clustering, we identified 9,613 clusters comprising both mapped and unmapped sequences (Additional file 5). Among them, only 7 clusters in amp1, 3 in amp2, 3 in amp3, 8 in amp4, 2 in amp5, 4 in amp6, and 9 in amp7&8 met the thresholds defined for this study (Figures 5, 6 and 7; Additional file 6). For each amplicon, there was a single major cluster that contained the majority (>45%) of reads, along with one or more minor cluster(s), each with a relatively small number of reads. As the clustering analysis applied a 99% identity threshold, larger deletions (> ~20 bp) influenced the resulting clusters much more than did shorter deletions and SNVs. The proportion of reads in each cluster varied across genome location, sampling time point, and host species. The 4wpc_Trout and 8wpc_Trout experimental groups had a significantly greater proportion of reads in some minor clusters than did the other experimental groups (Figures 5, 6 and 7; Additional file 6). This was most prominent for Amp7&8_cluster2 and Amp7&8_cluster3 for 8wpc_Trout (Figure 7). Most of the minor clusters predominantly exhibited frameshift deletions; however, each cluster was composed of sequences with 99% identity, resulting in the practical coexistence of both in-frame and frameshift deletion reads. In addition, in some raw clusters that did not pass the threshold, sequence inversion, transposition, insertion, and deletion were observed (Additional file 5).

### Nonmetric multidimensional scaling (NMDS) analysis of variation between experimental groups

**Figure 8:**
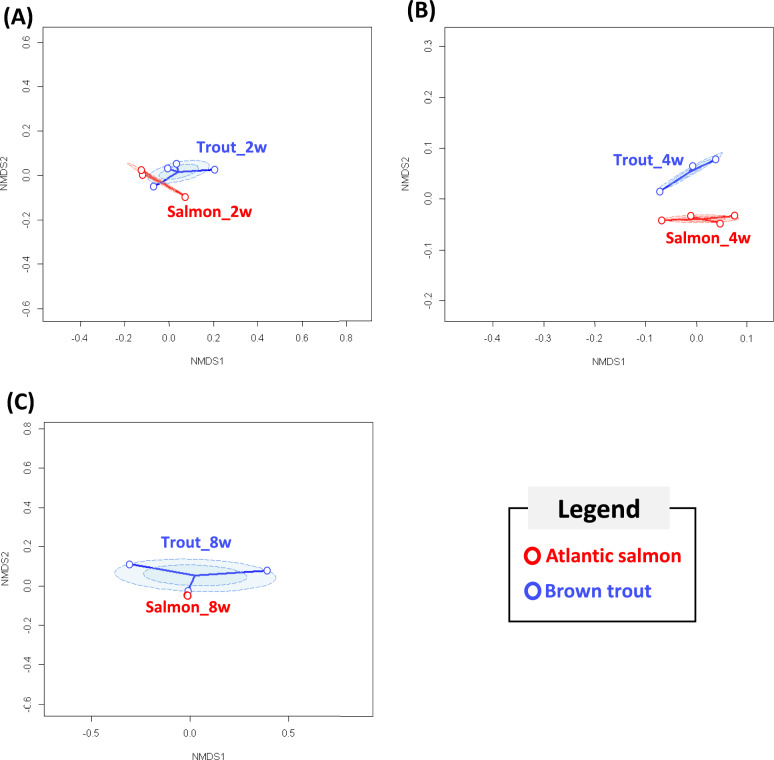
**Nonmetric multidimensional scaling (NMDS) plot NMDS plots.** generated from the read proportions of the 36 clusters from the amplicons amp1 to amp7&8 identified in this study. The distances on the plot reflect the similarities in the proportions of all clusters. Points closer together indicate a higher degree of similarity in cluster proportions, while points farther apart represent lower similarity. Figure 8A–C depict the comparisons between different species (salmon in red and trout in blue) at 2- (2wpc_Salmon vs 2wpc_Trout), 4- (4wpc_Salmon vs 4wpc_Trout), and 8-wpc (8wpc_Salmon vs 8wpc_Trout), respectively. The ellipses indicate confidence limits of 0.25 (darker red or blue) and 0.5 (lighter red or blue) within the same group.

NMDS analysis was used to analyse the variation (dissimilarity) between the experimental groups. In the NMDS analysis, 36 dimensions (i.e., the number of clusters) were condensed into two dimensions where the distance between experimental groups (and specimens) in an NMDS plot indicates the degree of similarity. At two weeks post-challenge, the experimental groups partially overlapped, and each showed relatively little variation between specimens (Figure 8A). At four weeks post-challenge, the experimental groups no longer overlapped but still showed relatively little variation between specimens (Figure 8B). At eight weeks post-challenge, the experimental groups were again partially overlapping but showed a distinct difference in variation between specimens (Figure 8C).

### Visualization of selected mutations in the spike protein

**Figure 9:**
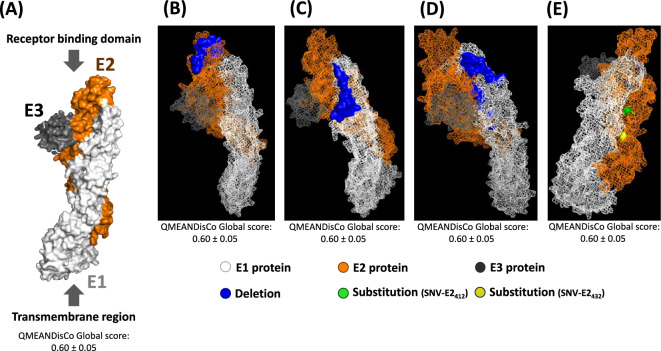
**Visualization of the locations of selected deletions and SNVs in the SAV3 spike protein**. A 3D structural model of the SAV3 spike protein consisting of the E1, E2 and E3 subunits was constructed via homology modelling and visualized. **A** Space-filling model of the SAV3 spike protein, which is a trimeric protein that includes E1 (white), E2 (orange), and E3 (gray). **B**, **C** and **D** The deletions identified in Amp6_cluster2, Amp7&8_cluster2, and Amp7&8_cluster3, respectively, are highlighted in blue. E Nonsynonymous minor SNVs (E2_412_ and E2_432_) are highlighted in light green and yellow, respectively. Comprehensive views of the entire 3D structures from various orientations are available in Additional file 6. The QMEANDisCo global score shown in Figure A-E gives an overall model quality measurement between 0 and 1, where higher numbers indicate higher expected quality.

A homology model of the SAV spike protein was constructed using SWISS-MODEL, and the model was subsequently used to visualize the location of selected mutations (Figure 9). Amp6_cluster2, Amp78_cluster2, and amp78_cluster3 exceeded a mean proportion of reads of 10% in at least one experimental group, showing statistically significant differences. The consensus sequences from both clusters are frameshift deletions located at the apical region of the spike protein. However, in reality, reads containing both in-frame and frameshift deletions coexist (Figures 8B–D). The major nonsynonymous SNVs identified in the SAV spike protein are highlighted in green and yellow in Figure 9E and Additional file 7. The QMEANDisCo global score, ranging from 0 to 1, expresses the quality of a predicted model [53]. Higher QMEANDisCo scores indicate better quality and accuracy in the predicted protein structure. While the acceptable range for the QMEANDisCo global score may vary depending on the types of predicted proteins, a score above 0.50 generally implies that the predicted model is likely acceptable based on the established threshold [54]. The predicted SAV spike protein model based on the 2wpc_consensus sequence had a QMEANDisCo global score of 0.60 ± 0.05, which is comparable to that of other models of alphavirus spike proteins deposited (e.g., Q5WQY5; Chikungunya virus- 0.65 ± 0.05 QMEANDisCo global score). The deletions (Amp6_cluster2, Amp78_cluster2, Amp78_cluster2) and nonsynonymous mutations did not affect the QMEANDisCo global score, as they showed the same values.

## Discussion

In the present study, we used the Nanopore long-read sequencing platform to sequence the salmonid alphavirus-3 (SAV3) genome from tissue samples collected from Atlantic salmon and brown trout at various time points during a virus challenge experiment. The primary source of SAV3 infection in cohabitants was the shedder fish. SAV3 sequences from the 2wpc_Salmon experimental group were analysed and used as a reference genome for the remaining experimental time points. The cohabitation challenge applied in this study has both advantages and disadvantages as a method for investigating SAV3 variants. The advantage of the cohabitation model is that it accurately replicates the actual route of waterborne SAV3 infection. However, cohabitation challenges also have potential limitations regarding two parameters: the actual dose of SAV3 to which cohabitant fish are exposed and the exact timing of their initial infection. These potential limitations should be noted when considering the population diversity of sequences within quasispecies at different time points post-infection.

Among the major nonsynonymous SNV-alleles, only two (SNV-nsP2_1672_-T/C and SNV-E2_1187_-T/C) were found in more than one fish. Among them, the SNV-E2_1187_-T/C, located within the spike protein, represented a nonsynonymous mutation that converts valine to alanine. This valine-to-alanine substitution may significantly influence viral fitness, leading to notable phenotypic changes. Interestingly, Tsetsarkin et al. [55] investigated the impact of an alanine-to-valine mutation at position 226 in the E1 fusion protein of Chikungunya virus (CHIKV). Compared with yellow fever mosquitos (*Ae. aegypti*), CHIKV with an alanine at this position (E1-226A) showed relatively rapid infection and an increased ability to infect Asian tiger mosquitos (*Ae. albopictus*). Conversely, CHIKV with valine at this position (E1-226 V) was significantly better at infecting yellow fever mosquitos. This study highlights how a single substitution can significantly alter the phenotypic characteristics of alphaviruses. Among several minor SNV-alleles identified between the experimental groups, only SNV-E2_412_-T/C was consistently and significantly more abundant in the trout experimental group and exhibited a distinct increase over time. At another site, two minor SNV-alleles (SNV-E2_432_-G/C and SNV-E2_432_-G/T) that both led to an E (glutamic acid) to D (aspartic acid) aa change also increased in SNV-allele_freq_ over time in the trout experimental group, but this increase was not statistically significant. In general, SNVs could alter viral tropism towards different hosts. The E2 protein is one of the three glycoproteins that makes up the SAV spike protein and is one of the structural proteins where most immunogenic epitopes are located [56, 57]. Karlsen et al. [58] observed the influence of a mutation at position E2_206_, from proline (E2_206p_) to serine (E2_206s_), which is located in the receptor binding site. The authors found that viral growth and replication differed significantly between these mutants. The E2_206s_ mutant also reverted to E2_206p_ when the virus was inoculated into a cell line (BF2), indicating that SAV3 may adapt to its host and environment. In the present study, the minor SNVs (E2_412_ and E2_432_) identified in the E2 gene are located in the middle of the spike protein rather than in the receptor binding site. Hence, the effect of these nonsynonymous mutations is likely less pronounced/direct than that of the variant observed in the study by Karlsen et al. [58]. On the other hand, most deletion mutations identified from minor clusters in the spike protein (Amp6_cluster2, Amp7&8_cluster2, and Amp7&8_cluster3) are located in a region that faces outwards from the viral membrane. Deletions in these regions could influence cellular tropism. In addition, introduction of minor SNV-nsP2_486_ may lead to the introduction of premature stop codons (TAG and TAA). Given that nonstructural proteins such as nsP2 regulate viral RNA synthesis, premature stop codons will result in a defective viral polyprotein unable to perform its role in viruses.

In the cluster analysis, the reads in each identified cluster had at least 99% sequence identity. Given that the genetic identity among SAV subtypes ranges from ~86–96% [3], we used the threshold of 99% sequence identity in the cluster analyses to allow the study of intrasubtype variation. If, in contrast, a threshold lower than ~96% sequence identity had been used, the cluster analysis would not have been able to differentiate between SAV subtypes. Since the amplicons (and hence the reads) had an average length of approximately 2000 bp, the clusters, on average, differed from each other in at least 20 nucleotides. Using these threshold conditions inadvertently led to all the identified clusters being predominantly defined by larger deletions. When the reads in each identified cluster were “merged” into a defining consensus sequence, these deletions mostly led to a shift in the reading frame. This would suggest that these deletion-defined clusters should be considered nonproductive dead ends. It should be noted, however, that among the reads in these clusters, there were sequences with in-frame deletions that, in principle, could retain (some) functionality. Similarly, Gallagher et al. [17] identified many deletion mutations based on nanopore sequencing, and ~34% of deletions did not disrupt the protein-coding frame (in-frame mutation), which leaves open the possibility that not all observed deletions result in defective viral particles. In addition, the sizes of the complete SAV genomes varied slightly (SAV1 (AJ316244.1; 11,919 bp), SAV2 (AJ316246.1; 11,900 bp), SAV3 (KC122926.1; 11,887 bp), SAV4 (MH708651.1; 11,762 bp), SAV5 (MH708650.1; 11,804 bp), and SAV6 (MH238448.1; 11,726 bp)). This difference may ultimately stem from the frequent occurrence of deletion mutations in SAV. Overall, the cluster analysis of each of the 8 amplicons revealed little directional development (i.e., adaptation) at different sampling time points or between fish species. The only exception was for amplicons 1 and 7/8, where the frequency of some minor clusters increased for brown trout at 8 wpc. 

NMDS analysis integrating the cluster data over all eight amplicons indicated that late in infection, SAV3 genomes from brown trout had higher levels of variation than did SAV3 genomes from salmon. At the first sampling time point (2wpc), little difference was observed in the NMDS plot. By 4 wpc, the experimental groups had similar levels of variation but were still separated in the NMDS plot. In contrast, the groups overlapped at 8 wpc, but the brown trout experimental group showed distinctly more variation. Considering the distinct kinetics observed between salmon and trout at 8 wpc, the susceptibility of brown trout to SAV3 may be lower than that of other trout species. The observed higher variation in brown trout could be interpreted as the SAV3 exploring the virus fitness landscape in a host to which it is not well adapted.

In conclusion, this study provides insight into the genetic variation in SAV3 in infected fish, revealing mostly random variation with no development in SNV_freq_ during the experiment. Nevertheless, a few specific variants, such as SNV-E2_412_ and SNV-E2_432_, increased in frequency with time, potentially showing viral adaptation to trout. We believe that this approach and bioinformatics pipeline will be useful for studies of viral variation and evolution.

## Supplementary Information

**Additional file 1. Basic characteristics of the reads and data used for sequencing and read mapping of the reference genome.**

**Additional file 2. Primers used in this study.**

**Additional file 3.The Ct values (mean ± SD) determined by RT-qPCR targeting the SAV3 nsP1 gene in the samples sequenced in this study.**

**Additional file 4. The frequency of minor SNVsin the experimental groups.** A total of 7 SNVs were identified as minor, as they had an SNV_freq_ between 5 and 60% in at least one experimental group. For each minor SNV, the table shows the frequency observed in the experimental groups and the results of Welch’s t test comparison of frequencies in the experimental groups and the 2wpc_Salmon consensus genome.

**Additional file 5. All the raw de novo clusters identified in this study.**

**Additional file 6. Consensus nucleotide sequences of clusters that passed the threshold in this study.**

** Additional file 7. Visualization of the locations of selected deletions and SNVs in the SAV3 spike protein.** A 3D structural model of the SAV3 spike protein consisting of the E1, E2 and E3 subunits was constructed via homology modelling and visualized in videos. (A) Space-filling model of the SAV3 spike protein, shown as a 12-meric protein including four E1 subunits (white), four E2 subunits (orange), and four E3 subunits (gray). (B, C and D) The deletions identified in Amp6_cluster2, Amp7&8_cluster2, and Amp7&8_cluster3, respectively, are highlighted in blue. (E) Nonsynonymous major SNVs (SNV-E2_1187_ and SNV-E1_1321_) are highlighted in green and purple, and two minor SNVs (SNV-E2_412_ and SNV-E2_432_) are shown in cyan and yellow.

## Data Availability

The datasets used in this study are available from the corresponding author upon reasonable request.
