# Supplementary material for: Nanopore sequencing provides snapshots of the genetic variation within salmonid alphavirus-3 (SAV3) during an ongoing infection in Atlantic salmon (Salmo salar) and brown trout (Salmo trutta)
Source: Vet Res. 2024 Sep 3;55:106. doi: 10.1186/s13567-024-01349-z (PMC11373506; doi:10.1186/s13567-024-01349-z)
Supplement: Supplementary file 1 — Additional file 1. Basic characteristics of the reads and data used for sequencing and read mapping of the reference genome. [file 13567_2024_1349_MOESM1_ESM.docx]

**Additional file 1. Basic characteristics of reads and data for sequencing read mapping of the reference genome.**

| **wpc** | **Experimental**  **Group** | **species** | **Sample ID** | **Number of raw reads^1^** | **Number of unmapped reads^2^** | **Uniquely mapped^3^** | **Percent of mapped reads^4^** |
| --- | --- | --- | --- | --- | --- | --- | --- |
| **2** | **2wpc**  **Salmon** | **Salmon**  **(*n* = 3)** | S_2w_1 | 4246 | 1075 | 3171 | 75% |
|  |  |  | S_2w_2 | 4630 | 2465 | 2165 | 47% |
|  |  |  | S_2w_3 | 3267 | 562 | 2705 | 83% |
|  | **2wpc**  **Trout** | **Trout**  **(*n* = 4)** | T_2w_1 | 5527 | 3549 | 1978 | 36% |
|  |  |  | T_2w_2 | 4386 | 2824 | 1562 | 36% |
|  |  |  | T_2w_3 | 5914 | 3329 | 2585 | 44% |
|  |  |  | T_2w_4 | 4560 | 1734 | 2826 | 62% |
| **4** | **4wpc**  **Salmon** | **Salmon**  **(*n* = 4)** | S_4w_1 | 4310 | 2478 | 1832 | 43% |
|  |  |  | S_4w_2 | 5858 | 3596 | 2262 | 39% |
|  |  |  | S_4w_3 | 3371 | 1232 | 2139 | 63% |
|  |  |  | S_4w_4 | 5398 | 3222 | 2176 | 40% |
|  | **4wpc**  **Trout** | **Trout**  **(*n* = 4)** | T_4w_1 | 5901 | 3754 | 1265 | 25% |
|  |  |  | T_4w_2 | 3619 | 2753 | 866 | 24% |
|  |  |  | T_4w_3 | 3746 | 2208 | 538 | 14% |
|  |  |  | T_4w_4 | 4950 | 3970 | 980 | 20% |
| **8** | **8wpc**  **Salmon** | **Salmon**  **(*n* = 4)** | S_8w_1 | 3534 | 1413 | 2121 | 60% |
|  |  |  | S_8w_2 | 4006 | 1792 | 2214 | 55% |
|  |  |  | S_8w_3 | 6552 | 1953 | 4599 | 70% |
|  |  |  | S_8w_4 | 4678 | 1990 | 2688 | 57% |
|  | **8wpc**  **Trout** | **Trout**  **(*n* = 3)** | T_8w_1 | 4032 | 1920 | 2112 | 52% |
|  |  |  | T_8w_2 | 3554 | 1734 | 1820 | 51% |
|  |  |  | T_8w_3 | 1722 | 1008 | 714 | 41% |

^1^ Total number of reads by Oxford nanopore after duplex basecalling.

^2^ Total number of unmapped reads onto the reference genome.

^3^ Total number of reads that were uniquely mapped.

^4^ Percent of reads that were mapped onto the reference genome.
