# Supplementary material for: Nanopore sequencing provides snapshots of the genetic variation within salmonid alphavirus-3 (SAV3) during an ongoing infection in Atlantic salmon (Salmo salar) and brown trout (Salmo trutta)
Source: Vet Res. 2024 Sep 3;55:106. doi: 10.1186/s13567-024-01349-z (PMC11373506; doi:10.1186/s13567-024-01349-z)
Supplement: Supplementary file 2 — Additional file 2. Primers used in this study. [file 13567_2024_1349_MOESM2_ESM.docx]

**Additional file 2.** **Primers used in this study.**

| **Amplicon** | **Primer** | **sequence** |
| --- | --- | --- |
| **amp1** | sav_amp1_F | **5'-TCCAAAAGCATACATATATCAATGATGCA-3'** |
|  | sav_amp1_R | **5'-GCCCTTGTGGACATACTGCATC-3'** |
| **amp2** | sav_amp2_F | **5'-GCCAACCCTAGTTACATCGCAG-3'** |
|  | sav_amp2_R | **5'-GCGTGGATTGGCGATCTCATAG-3'** |
| **amp3** | sav_amp3_F | **5'-CCTGTTGGGCAGTTACAAGCAG-3'** |
|  | sav_amp3_R | **5'-TGGAACTTCGTGCCGACAAAAA-3'** |
| **amp4** | sav_amp4_F | **5'-GTCACCATCTATTGCTTGGCCA-3'** |
|  | sav_amp4_R | **5'-ACTTGGGACTGTATAAGCGGCT-3'** |
| **amp5** | sav_amp5_F | **5'-TTTCCTAAGCCCAGGTACTCCG-3'** |
|  | sav_amp5_R | **5'-AGATCCTTACCTCCTTTCCGGG-3'** |
| **amp6** | sav_amp6_F | **5'-CTCGAGGACAAGTACAGCCGTA-3'** |
|  | sav_amp6_R | **5'-TTGCTGTGGAAACCAAGGTTCC-3'** |
| **amp7** | sav_amp7_F | **5'-TTTACGTGTGAGGAGCCGGTTC-3'** |
|  | sav_amp7_R | **5'-TGCCGAACAGATCTCTACCTCG-3'** |
| **amp8** | sav_amp8_F | **5'-TGTTAGGTACGCATCTGGGGAC-3'** |
|  | sav_amp1_R | **5'-ACTTCTTCACCACGCAGTAGGT-3'** |
