# Supplementary material for: Nanopore sequencing provides snapshots of the genetic variation within salmonid alphavirus-3 (SAV3) during an ongoing infection in Atlantic salmon (Salmo salar) and brown trout (Salmo trutta)
Source: Vet Res. 2024 Sep 3;55:106. doi: 10.1186/s13567-024-01349-z (PMC11373506; doi:10.1186/s13567-024-01349-z)
Supplement: Supplementary file 3 — Additional file 3.The Ct values (mean ± SD) determined by RT-qPCR targeting the SAV3 nsP1 gene in the samples sequenced in this study. [file 13567_2024_1349_MOESM3_ESM.docx]

**Additional file 3. The Ct values (mean ± SD) determined by RT-qPCR targeting SAV3 nsP1 gene of samples sequenced in this study.**

| Species | **Weeks post-challenge (wpc)** | | |
| --- | --- | --- | --- |
|  | 2 | 4 | 8 |
| Salmon | 28.9 ± 6.3^ab^ | 22.6 ± 3.9^c^ | 26.8 ± 0.4^a^ |
| Trout | 25.9 ± 4.0^bc^ | 21.9 ± 0.8^c^ | 33.4 ± 1.0^bc^ |

+ Superscripts with different alphabetic letters indicate the statistically significant differences based on ANOVA Duncan multiple range test for all experimental groups (salmon and trout).
