# Supplementary material for: Nanopore sequencing provides snapshots of the genetic variation within salmonid alphavirus-3 (SAV3) during an ongoing infection in Atlantic salmon (Salmo salar) and brown trout (Salmo trutta)
Source: Vet Res. 2024 Sep 3;55:106. doi: 10.1186/s13567-024-01349-z (PMC11373506; doi:10.1186/s13567-024-01349-z)
Supplement: Supplementary file 4 — Additional file 4. The frequency of minor SNVsin the experimental groups. A total of 7 SNVs were identified as minor, as they had an SNVfreq between 5 and 60% in at least one experimental group. For each minor SNV, the table shows the frequency observed in the experimental groups and the results of Welch’s t test comparison of frequencies in the experimental groups and the 2wpc_Salmon consensus genome. [file 13567_2024_1349_MOESM4_ESM.docx]

**Additional file 4. The frequency of minor SNVs in the experimental groups.** A total of 7 SNVs were identified as minor, as they had an SNV_freq_ between 5 and 60% in at least one experimental group. For each minor SNV, the table shows the frequency observed in the experimental groups and results of Welch’s t-test comparison of frequencies in experimental groups and the 2wpc_Salmon consensus genome.

| **Gene^1)^** | **Minor SNV**^2)^ | **Minor SNV-allele** | **Amino acid^3)^** | | **Salmon** | **Salmon** | | **Trout** | | |
| --- | --- | --- | --- | --- | --- | --- | --- | --- | --- | --- |
|  |  |  | WT | Variant | 2wpc^4)^ | 4wpc^5)^ | 8wpc^5)^ | 2wpc^5)^ | 4wpc^5)^ | 8wpc^5)^ |
| **nsP2** | **nsP2_486_** | nsP2_486_-C/A | TAC (Y) | TA**A** (*****) | 0.0±0.0 | **2.6±0.8**  **(0.008)** | 5.1±3.2  (0.050) | 0.4±0.7  (0.391) | **3.1±1.5**  **(0.023)** | 0.0±0.0  (-) |
|  |  | nsP2_486_-C/T |  | TA**T** (Y) | 0.0±0.0 | 0.6±0.6  (0.131) | 0.1±0.2  (0.391) | 0.0±0.0  (-) | 0.3±0.4  (0.183) | 0.0±0.0  (-) |
|  |  | nsP2_486_-C/G |  | TA**G** (*****) | 0.0±0.0 | **3.2±0.5**  **(0.001)** | 5.0±3.3  (0.057) | 0.4±0.7  (0.391) | **4.2±2.2**  **(0.033)** | 0.0±0.0  (-) |
|  |  | **Minor SNV_freq_** | | | 0.0±0.0 | **6.5±1.6**  **(0.004)** | 10.2±6.4  (0.050) | 0.7±1.4  (0.391) | **7.7±3.8**  **(0.026)** | 0.0±0.0  (-) |
|  | **nsP2_505_** | nsP2_505_-G/A | GCG (A) | **A**CG (**T**) | 0.8±1.3 | 4.1±3.1  (0.115) | 3.4±2.3  (0.117) | 1.9±1.8  (0.391) | **4.7±2.3**  **(0.037**) | 0.2±0.3  (0.513) |
|  |  | nsP2_505_-G/T |  | **T**CG (**S**) | 0.4±0.7 | 4.2±2.5  (0.054) | 3.7±2.9  (0.104) | 1.6±1.8  (0.291) | **5.8±3.3**  **(0.042)** | 0.2±0.3  (0.630) |
|  |  | nsP2_505_-G/C |  | **C**CG (**P**) | 0.4±0.7 | **4.8±2.3**  **(0.024)** | 3.7±2.9  (0.104) | 1.6±1.8  (0.291) | **5.2±2.9**  **(0.040)** | 0.2±0.3  (0.630) |
|  |  | **Minor SNV_freq_** | | | 1.5±2.6 | 13.1±7.8  (0.053) | 10.8±8.1  (0.103) | 5.0±5.4  (0.313) | **15.7±8.4**  **(0.037)** | 0.5±0.8  (0.568) |
|  | **nsP2_531_** | nsP2_531_-A/T | ACA (T) | AC**T** (T) | 0.0±0.0 | 1.1±0.9  (0.097) | 3.4±2.5  (0.072) | 0.2±0.3  (0.391) | 2.4±1.8  (0.083) | 0.0±0.0  (-) |
|  |  | nsP2_531_-A/G |  | AC**G** (T) | 0.0±0.0 | 1.9±2.1  (0.164) | **4.1±2.1**  **(0.029)** | 4.7±4.7  (0.137) | 5.6±4.3  (0.081) | 0.3±0.6  (0.423) |
|  |  | nsP2_531_-A/C |  | AC**C** (T) | 0.0±0.0 | 1.7±1.8  (0.157) | **3.0±1.9**  **(0.048)** | 0.9±1.4  (0.283) | 3.7±3.2  (0.101) | 0.0±0.0  (-) |
|  |  | **Minor SNV_freq_** | | | 0.0±0.0 | 4.7±4.6  (0.135) | **10.6±6.3**  **(0.044)** | 5.8±5.4  (0.119) | 11.7±9.3  (0.087) | 0.3±0.6  (0.423) |
| **nsP3** | **nsP3_1190_** | nsP2_1190_-C/A | TCC (S) | T**A**C (**Y**) | 0.0±0.0 | 1.2±2.3  (0.367) | 0.0±0.0  (-) | 0.0±0.0  (-) | 0.1±0.3  (0.391) | 0.1±0.1  (0.423) |
|  |  | nsP2_1190_-C/T |  | T**T**C (**F**) | 0.0±0.0 | 2.2±3.8  (0.326) | 0.0±0.0  (-) | **5.5±1.5**  **(0.005)** | 1.2±1.7  (0.265) | 0.2±0.4  (0.423) |
|  |  | nsP2_1190_-C/G |  | T**G**C (**C**) | 0.0±0.0 | 1.4±2.6  (0.351) | 0.0±0.0  (-) | 0.0±0.0  (-) | 0.1±0.3  (0.391) | 0.1±0.1  (0.423) |
|  |  | **Minor SNV_freq_** | | | 0.0±0.0 | 4.9±8.7  (0.344) | 0.0±0.0  (0.050) | **5.5±1.5**  **(0.005)** | 1.4±1.7  (0.203) | 0.4±0.6  (0.499) |
| **nsP4** | **nsP4_948_** | nsP4_948_-C/A | GCC (A) | GC**A** (A) | 0.2±0.4 | 3.0±2.2  (0.08) | 5.6±6.6  (0.2) | 0.7±0.9  (0.377) | 6.4±8.1  (0.221) | 0.4±0.7  (0.723) |
|  |  | nsP4_948_-C/T |  | GC**T** (A) | 0.0±0.0 | 0.3±0.6  (0.391) | 1.2±1.1  (0.115) | 0.1±0.3  (0.391) | 0.0±0.0  (-) | 0.5±0.8  (0.423) |
|  |  | nsP4_948_-C/G |  | GC**G** (A) | 0.0±0.0 | 1.3±1.8  (0.254) | 1.9±1.8  (0.133) | 0.1±0.3  (0.391) | 0.0±0.0  (-) | 0.5±0.8  (0.423) |
|  |  | **Minor SNV_freq_** | | | 0.2±0.4 | 4.6±3.7  (0.099) | 8.7±9.4  (0.170) | 1.0±1.2  (0.291) | 6.4±8.1  (0.221) | 1.3±2.3  (0.499) |
| **E2** | **E2_412_** | E2_412_-T/A | TTC (F) | **A**TC (**I**) | 0.1±0.2 | 0.2±0.5  (0.682) | 0.2±0.2  (0.762) | 0.5±0.9  (0.522) | 0.5±1.0  (0.512) | 0.4±0.7  (0.578) |
|  |  | E2_412_-T/G |  | **G**TC (**V**) | 0.7±0.7 | 0.9±1.1  (0.82) | 0.2±0.3  (0.357) | 1.1±0.9  (0.631) | 0.5±0.6  (0.707) | 0.4±0.7  (0.562) |
|  |  | E2_412_-T/C |  | **C**TC (**L**) | 2.2±3.2 | 4.5±4.2  (0.451) | 0.2±0.3  (0.397) | **9.4±3.1**  **(0.038)** | **9.9±4.5**  **(0.048)** | 21.7±37.6  (0.464) |
|  |  | **Minor SNV_freq_** | | | 3.1±3.9 | 5.7±5.1  (0.483) | 0.6±0.8  (0.386) | **10.9±3.0**  **(0.047)** | 10.9±5.2  (0.071) | 22.5±39.0  (0.480) |
|  | **E2_432_** | E2_432_-G/A | GAG (E) | GA**A** (E) | 0.3±0.3 | 0.6±0.8  (0.579) | 0.5±0.8  (0.675) | 0.8±0.4  (0.125) | 1.1±0.6  (0.071) | 0.2±0.3  (0.125) |
|  |  | E2_432_-G/T |  | GA**T** (**D**) | 0.6±0.5 | 2.0±1.8  (0.212) | 0.0±0.0  (0.186) | 2.3±2.3  (0.226) | **2.4±1.2**  **(0.047)** | 10.8±18.7  (0.226) |
|  |  | E2_432_-G/C |  | GA**C** (**D**) | 1.8±1.6 | 3.6±2.9  (0.353) | 0.1±0.3  (0.219) | 4.5±4.5  (0.327) | 7.3±3.8  (0.055) | 11.4±19.7  (0.327) |
|  |  | **Minor SNV_freq_** | | | 2.7±2.4 | 6.1±5.2  (0.303) | 0.7±0.8  (0.269) | 7.7±7.2  (0.266) | **10.9±5.2**  **(0.044)** | 22.3±38.7  (0.472) |

1) the gene in which the SNV is situated, 2) details of the SNV, 3) resulting change in amino acid, i.e. from WT (2wpc_Salmon consensus genome) to variant (changes shown in red), 4) Frequency of the minor SNV in the 2wpc_Salmon consensus genome, 5) Frequency of the minor SNV in the experimental group. Numbers inside brackets shows p-values from Welch's t-test comparing the SNV frequency in the experimental group with that of the 2wpc_Salmon consensus genome (bold letters indicate P-values less than 0.05). SNVs highlighted with a background color range from yellow to red, representing SNVfreq values from 5% (yellow) to the highest value (red), with the color intensifying progressively as the values increase.
