# Supplementary figures and images for: Nanopore sequencing provides snapshots of the genetic variation within salmonid alphavirus-3 (SAV3) during an ongoing infection in Atlantic salmon (Salmo salar) and brown trout (Salmo trutta)
Source: Vet Res. 2024 Sep 3;55:106. doi: 10.1186/s13567-024-01349-z (PMC11373506; doi:10.1186/s13567-024-01349-z)

## Slide 1
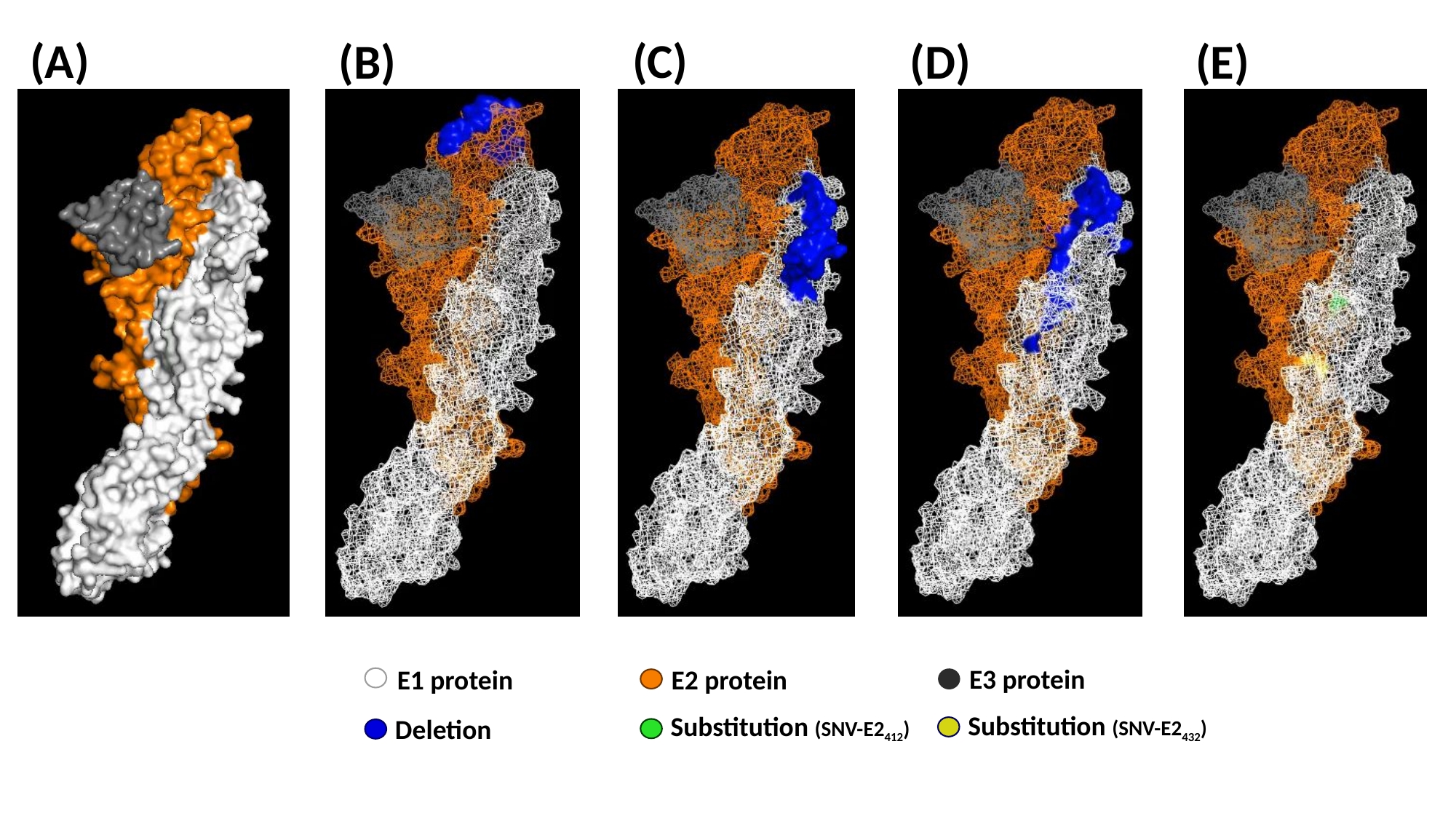

(C)
(A)
(B)
(E)
(D)
E1 protein
E3 protein
E2 protein
Substitution (SNV-E2412)
Substitution (SNV-E2432)
Deletion

Supplement: Supplementary file 7 — Additional file 7. Visualization of the locations of selected deletions and SNVs in the SAV3 spike protein. A 3D structural model of the SAV3 spike protein consisting of the E1, E2 and E3 subunits was constructed via homology modelling and visualized in videos. (A) Space-filling model of the SAV3 spike protein, shown as a 12-meric protein including four E1 subunits (white), four E2 subunits (orange), and four E3 subunits (gray). (B, C and D) The deletions identified in Amp6_cluster2, Amp7&8_cluster2, and Amp7&8_cluster3, respectively, are highlighted in blue. (E) Nonsynonymous major SNVs (SNV-E21187 and SNV-E11321) are highlighted in green and purple, and two minor SNVs (SNV-E2412 and SNV-E2432) are shown in cyan and yellow. [file 13567_2024_1349_MOESM7_ESM.pptx]
